# A rice transient assay system identifies a novel domain in NRR required for interaction with NH1/OsNPR1 and inhibition of NH1-mediated transcriptional activation

**DOI:** 10.1186/1746-4811-8-6

**Published:** 2012-02-21

**Authors:** Mawsheng Chern, Wei Bai, Wing Hoi Sze-To, Patrick E Canlas, Laura E Bartley, Pamela C Ronald

**Affiliations:** 1 Department of Plant Pathology, University of California, Davis, Davis, CA 95616, USA; 2 College of Life Sciences, Inner Mongolia Agricultural University., Huhhot 010018, China; 3 Department of Botany and Microbiology, University of Oklahoma, Norman, OK 73019, USA

## Abstract

**Background:**

Arabidopsis NPR1 is a master regulator of systemic acquired resistance. NPR1 binds to TGA transcription factors and functions as a transcriptional co-activator. In rice, NH1/OsNPR1 functions to enhance innate immunity. NRR disrupts NH1 function, when over-expressed.

**Results:**

We have established a rice transient protoplast assay to demonstrate that NH1 is a transcriptional co-activator and that NRR represses NH1-mediated activation. We identified three NRR homologues (RH1, RH2, and RH3). RH1 and RH3, but not RH2, also effectively repress NH1-mediated transcriptional activation. NRR, RH1, RH2, and RH3 share sequence similarity in a region beyond the previously identified NPR1-interacting domain. This region is required for strong interaction with NH1. A double point mutation, W66A/F70A, in this novel NH1-interacting domain severely reduces interaction with NH1. Mutation W66A/F70A also greatly reduces the ability of NRR to repress NH1-mediated activation. RH2 carries a deviation (amino acids AV) in this region as compared to consensus sequences (amino acids ED) among NRR, RH1, and RH3. A substitution (AV to ED) in RH2 results in strong binding of mutant RH2ED to NH1 and effective repression of NH1-mediated activation.

**Conclusions:**

The protoplast-based transient system can be used to dissect protein domains associated with their functions. Our results demonstrate that the ability of NRR and its homologues to repress NH1-mediated transcriptional activation is tightly correlated with their ability to bind to NH1. Furthermore, a sequence is identified as a novel NH1-interacting domain. Importantly, this novel sequence is widely present in plant species, from cereals to castor bean plants, to poplar trees, to Arabidopsis, indicating its significance in plants.

## Background

Plants survive pathogen attack by employing various defense strategies, including strengthening of cell walls, the accumulation of phytoalexins, synthesis of salicylic acid (SA), and induction of pathogenesis-related (*PR*) genes. A hypersensitive response (HR) is often associated with the defense response and limits pathogen growth to the infected site. After an initial local infection, systemic acquired resistance (SAR) often occurs, which coordinately induces expression of a set of *PR *genes, leading to a long-lasting enhanced resistance against a broad spectrum of pathogens [1]. In dicots, like Arabidopsis and tobacco, SA and its synthetic analogs, 2,6-dichloroisonicotinic acid (INA), benzothiadiazole (BTH), and probenazole, are potent inducers of SAR [2-4]. In monocots, SAR can be induced by BTH in wheat [5] and by *Pseudomonas syringae *in rice [6]. BTH can also induce disease resistance in rice [7-9] and maize [10].

The *NPR1 *(also known as *NIM1 *and *SAI1*) gene is a key regulator of SA-mediated SAR in Arabidopsis [11-15]. Upon induction by SA, INA, or BTH, *NPR1 *expression levels are elevated [16]. *NPR1 *affects the SAR pathway downstream of the SA signal. Arabidopsis *npr1/nim1 *mutants are impaired in their ability to induce *PR *gene expression or to mount a SAR response even after treatment with SA or INA. *NPR1 *encodes a protein with a bipartite nuclear localization sequence and two protein-protein interaction domains: an ankyrin repeat domain and a BTB/POZ domain [16]. Nuclear localization of NPR1 is essential for its function [17]. During non-induced states, the NPR1 protein forms an oligomer and is excluded from the nucleus. Upon SAR induction, monomeric NPR1 emerges through redox changes, accumulates in the nucleus, and activates *PR *gene expression [18]. NPR1 also appears to modulate the cross talk between SA- and JA-dependent pathways; the antagonistic effect of SA on JA signaling requires NPR1, but not nuclear localization of the NPR1 protein [19]. In Arabidopsis, over-expression of *NPR1 *leads to enhanced disease resistance to both bacterial and oomycete pathogens [20]. In rice, over-expression of Arabidopsis *NPR1 *[21] or the rice orthologue *NH1 *[22] results in enhanced resistance to the pathogen *Xanthomonas oryzae *pv. *oryzae *(*Xoo*). Introduction of an extra copy of the paralogous gene *NH3 *in rice leads to enhanced resistance to *Xoo *and hyper-responsiveness to BTH treatment [23].

In search for proteins that mediate NPR1 function, several groups have identified TGA family members of basic-region leucine zipper (bZIP) transcription factors, both from Arabidopsis [24,25] and from rice [21], as NPR1 interacting proteins. The ankyrin repeats of NPR1 are necessary and sufficient for the interaction with TGA proteins [24]. The interaction between NPR1 and TGA proteins facilitates *in vitro *binding of the TGA proteins [25] and recruits them *in vivo *[26] to the SA-responsive promoters. *In vivo *interaction between NPR1 and a GAL4:TGA2 fusion (GAL4 DNA-binding domain fused to TGA2) protein leads to SA-mediated gene activation in Arabidopsis [27], supporting the notion that NPR1 binds to TGA2, which mediates transcriptional activation of downstream genes. The role of TGA proteins in mediating NPR1 function was further demonstrated by mutational analysis. The Arabidopsis triple knockout mutant *tga2tga5tga6 *blocks induction of *PR *gene expression and pathogen resistance [28]. TGA2, TGA5, and TGA6 function redundantly as negative regulators of *PR *genes before induction [28,29]. NPR1 functions as a transcriptional co-activator in a TGA2-NPR1 complex after SA treatment in a transient assay; this function requires the BTB/POZ domain and the oxidation of NPR1 Cys-521 and Cys-529 [29]. The BTB/POZ domain interacts with the repression domain of TGA2 to negate its function [30].

In Arabidopsis, another group of NIM1/NPR1 interacting proteins were identified and named NIMIN1-3, which share very limited sequence similarity [31]. A 10-amino-acid stretch, containing motif DXFFK, shared between NIMIN-1 and NIMIN-2 constitutes an NPR1 interacting domain [31]. NIMIN-1 and NIMIN-2 both contain putative nuclear localization signals. NIMIN-1 and NIMIN-3 share prolonged stretches of acidic amino acids and an almost identical stretch of 8 amino acids with unknown function. All three NIMIN proteins contain a short LXL repeat near the C-terminus. In rice, we have previously identified an NH1/OsNPR1 interactor, NRR, which shares very limited similarity with NIMIN2 in the NPR1 interacting domain identified by Weigel et al. [32]. Three NIMIN2-like proteins from tobacco were identified later as NPR1 interactors [33]. Over-expression of *NIMIN1 *in Arabidopsis compromises SAR [34]. Over-expression of NRR leads to super-susceptibility to *Xoo *and compromises *Xa21*-mediated resistance to *Xoo *in rice [32] and blocks SAR in Arabidopsis [35]. Knockout and RNA-silencing of *NIMIN1 *resulted in enhanced *PR-1 *gene expression after SA treatment, but no clear effects on disease resistance were observed [34].

We have previously shown that the rice NRR protein interacts with both the Arabidopsis NPR1 protein and the rice NH1 protein. We also showed that the NPR1-interacting domain in NRR is sufficient for strong interaction with NPR1 but not enough for rice NH1, suggesting another region in NRR required for strong interaction with NH1. NRR only shares limited similarity to Arabidopsis NIMIN2 in the NPR1-interacting domain and a short EAR (ERF-associated amphiphilic repression) [36] motif-like sequence (LDLNxxP) near the C-terminus [32].

Protoplast-based transient assay systems are powerful tools for research. We and others previously reported the use of a protoplast-based transient expression system [37,38]. To further explore NH1 and NRR functions and interaction, we have modified this system and used it to show that NH1 functions as a transcriptional co-activator and NRR represses this NH1-mediated activation. We found that the ability of NRR to repress NH1-mediated activation is completely correlated with its ability to interact with NH1. We identified a second region required for strong interaction with NH1. This region is conserved among rice NRR homologous proteins. Thus, NRR and homologues contain a novel domain for interaction with NH1. This sequence is also present in wheat, maize, sorghum, *Populus, Ricinus*, and Arabidopsis.

## Results

### Transient expression of a UAS-Luc reporter in rice protoplasts demonstrates transcriptional co-activator activity for NH1/OsNPR1

Our previously reported transient expression system used rice cultivar TP309. We have modified the system to take advantage of the superior genetic properties of Kitaake rice [39]. We also introduced the reporter construct UAS-Luc, which contains six copies of the Gal4 DNA binding site and a minimal TATA box driving the expression of the luciferase (Luc) gene. One of the UAS-Luc transgenic lines was adapted for the transient expression assay to facilitate detection. Rice protoplasts were prepared from 10-day old green seedlings grown in defined agar medium under sterile conditions. Upon transfection, another reporter plasmid Ubi-Gus, where the *Gus *gene is expressed from the constitutive maize *Ubi-1 *promoter, was included as reference. The experimental reporter activity is thus expressed as Luc activity normalized to Gus activity. The Luc reporter activity is directly dependent on the proteins binding to the UAS sites, namely the Gal4 DNA binding domain and its associated proteins.

Previous studies have shown that Arabidopsis NPR1 acts as a transcriptional co-activator in the presence of SA when transiently expressed in Arabidopsis [29]. We therefore first tested if the rice NH1 protein also acts as a transcriptional co-activator in our rice protoplast transient expression system. We generated two constructs, Gal4:rTGA2.1 and Gal4:rLG2, where two rice TGA family transcription factors rTGA2.1 and rLG2 that interact with NH1 [22] were fused to Gal4, by replacing their basic-leucine-zipper (bZIP) DNA binding domain with the Gal4 DNA binding domain. The *Ubi-1 *promoter was used to drive expression. The corresponding proteins Gal4:rTGA2.1 and Gal4:rLG2 serve to anchor to the UAS-Luc reporter. To test the activity of NH1, we generated an effector construct Ubi-NH1, where the *NH1 *cDNA is expressed from the *Ubi-1 *promoter.

**Figure 1:**
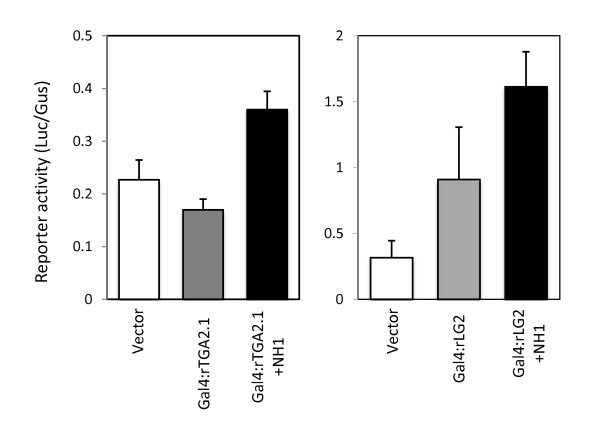
**Transient cell assay on transcriptional activation activity of rTGA2.1, rLG2, and NH1**. Protoplast cells were prepared from 10 days old green, transgenic rice seedlings, containing a UAS-Luc reporter. Protoplasts were transfected with combinations of plasmid constructs and Luc and Gus enzyme activities assayed after 20 h incubation at 28°C in growth chamber. The Ubi-Gus plasmid was included in all transfections and the Gus activity assayed for reference. In blanks, a Ubi-pUC plasmid was included to compensate for the amount of input DNA. rTGA2.1 and rLG2 are fused to the Gal4 DNA binding domain respectively, generating Gal4:rTGA2.1 and Gal4:rLG2. NH1 was expressed from the Ubi-NH1 construct. The UAS-Luc reporter activity is expressed as Luc/Gus. Each bar represents the average and standard deviation of three independent transfections.

As shown in the left panel of Figure 1, when the Gal4:rTGA2.1 construct was introduced into protoplasts, the expression level of the UAS-Luc reporter was modestly reduced, compared to the control which contained an empty vector (Ubi-pUC). This result is consistent with previous study on Arabidopsis TGA2, which shows that Arabidopsis TGA2 is a transcriptional repressor [29,30]. When the NH1 construct was also included, the reporter expression level went up by about two fold, rising above the background level, showing the co-activator activity of NH1. When the Gal4:rLG2 construct was introduced into protoplasts, the reporter expression was activated by about 2-3 fold (Figure 1, right panel). This result suggests that rLG2, which is a TGA family member most similar to the maize LG2 protein [21], carries an intrinsic activation domain and may function as a transcriptional activator. When NH1 was co-introduced, it further activated the reporter by roughly two fold, indicating that NH1 acts as a co-activator on both rTGA2.1 and rLG2.

### NRR acts as a transcriptional repressor in the transient assay system

We have previously reported that NRR is a negative regulator of disease resistance when over-expressed in rice [22] and Arabidopsis [35]. We have also identified an NPR1-interacting domain consisting of 25 amino acids (#28-52), which is similar to the NPR1-interacting domain identified in NIMIN proteins [31], and the region beyond the NPR1-interacting domain as required for strong interaction with rice NH1 in yeast two-hybrid [32]. Here, we tested to see if NRR affects NH1-mediated transcriptional activation. The *Ubi-1 *promoter was used to drive NRR expression. Gal4:rLG2 was chosen for further experimentation concerning the effects of NRR because of the relative ease of detection.

**Figure 2:**
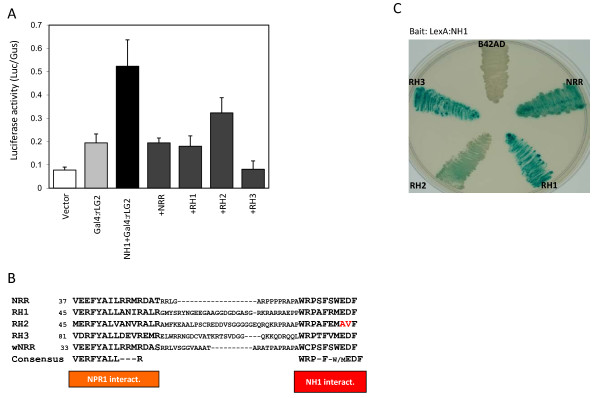
**Transient assay for effects of NRR, RH1, RH2, and RH3 on NH1-mediated activation**. (**A**) Protoplasts preparation and transfection were done as described in Figure 1. The Gal4:rLG2 and Ubi-NH1 constructs are as described above. NRR, RH1, RH2, and RH3 were expressed from the *Ubi-1 *promoter. Each bar represents the average and standard deviation of three replicates. (**B**) Sequence lineup of the NPR1-interacting domain and the NH1-interacting domain. Sequences within the NPR1 interacting and NH1-interacting domains of rice NRR, RH1, RH2, RH3, and wheat NRR ortholog (wNRR) are lined up. A consensus sequence is deduced. (**C**) Yeast two-hybrid assay. The NH1 protein is fused to the LexA protein (bait) and NRR, RH1, RH2, and RH3 are fused to the B42AD protein (prey). A positive interaction between the bait and the prey results in blue colors.

As shown in Figure 2A, When NH1 and Gal4:rLG2 are co-introduced into rice protoplasts, the UAS-Luc reporter expression is activated as before. Co-introduction with NRR completely neutralizes the NH1-mediated activation.

### NRR and its homologous proteins contain a second domain required for strong interaction with NH1

As amino acids 52 to 76 of NRR contain a domain required for strong interaction with NH1, we used this sequence and the previously identified NPR1-interacting domain sequence to search rice database for proteins that contain similar sequences. We found three other rice proteins that contain both putative domains. These three NRR homologous proteins are named RH1 (TIGR ID Os05g30500), RH2 (Os01g32460), and RH3 (Os01g32380) (for NRR Repressor Homologues). In addition, RH1 was also pulled out as an NPR1-interactor in our previous yeast two-hybrid screen. Figure 2B shows their sequence lineup within the two domains. Also included is the wheat ortholog of NRR (wNRR). Consensus sequences for the two domains are deduced from the five proteins: VERFYALLxxxR for the NPR1-interacting domain and WRPxFx[^W^/_M_]EDF for the putative NH1-interacting domain.

The ability of these rice proteins to interact with NH1 was tested. Figure 2C shows the results of a yeast two hybrid assay where blue colors indicate a positive interaction. The results show that RH1 and RH3 interact with NH1 as strongly as NRR, whereas RH2 only interacts weakly with NH1. The weak interaction of RH2 with NH1 may reflect its sequence deviation in the second domain, where it carries amino acids AV (highlighted in red in Figure 2B) in place of ED as in the consensus sequence. Nonetheless, the yeast two-hybrid protein-protein interaction results verify our observations that the two conserved regions are sufficient for strong interaction with NH1. Thus, NRR, RH1, RH2, and RH3 can indeed be classified as a family of NH1 interacting proteins. The newly identified NH1-interacting sequence (WRPxFx[^W^/_M_]EDF) represents a novel protein-protein interacting domain. BlastP searches on available databases in GenBank reveal that additional proteins with both the NPR1-interacting domain and the NH1-interacting domain are present in maize (gene ID 100274910), sorghum (ID 8065874), *Populus trichocarpa *(ID 7481426), and *Ricinus communis *(ID 8269866). A close examination of a sequence (PA/SFQPEDF) conserved between NIMIN1 and NIMIN3 identified by Weigel et al. (2001) also reveals similarity with the NH1-interacting sequence. No function for this conserved sequence in NIMIN1 and NIMIN3 has been identified. NIMIN2 does not contain this conserved sequence even though it is the one most similar to NRR among the three NIMIN proteins. The significance of this similarity between rice NRR and Arabidopsis NIMIN1 and NIMIN3 remains to be determined.

### RH1, RH2, and RH3 also repress NH1-mediated activation to various degrees

Since RH1, RH2, and RH3 interact with NH1, we tested if they also affect the transcriptional activation by NH1 in the protoplast cell transient system. The results are shown in Figure 2A. Like NRR, RH1 disrupts the activation by NH1, yielding expression levels similar to that of rLG2 alone. RH2 is less effective in repressing the NH1-mediated transcriptional activation, reducing the reporter expression only modestly. This result of RH2 correlates with its lower ability to interact with NH1 in yeast two-hybrid. RH3 reduces the reporter expression to a level even lower than that of NRR, indicating that RH3 may be a more effective repressor for NH1 than all other three proteins. Thus, rice NRR members all act as repressors of the NH1-mediated transcriptional activation, albeit to different degrees, in the transient cell assay system.

### Point mutations in the NH1-interacting domain diminish interaction with NH1

**Figure 3:**
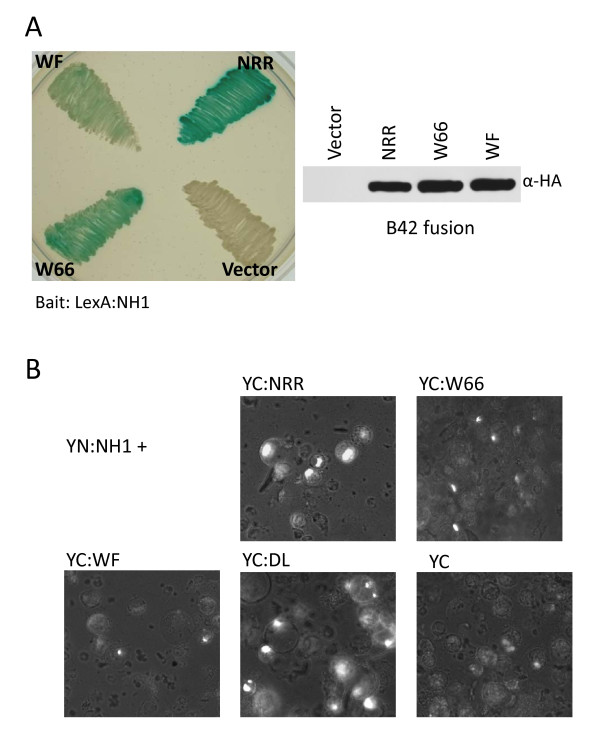
**Effects of point mutations on interaction of NRR with NH1**. (**A**) Yeast two-hybrid assay. The NH1 bait is fused to the LexA protein. NRR and mutants W66A and W66A/F70A are fused to the B42AD protein as prey. Blue colors indicate positive interactions. Protein was extracted from yeast cells containing constructs expressing LexA:NH1 plus B42AD (labeled vector), B42AD:NRR (NRR), B42AD:W66 (W66), or B42AD:WF (WF). Extracted protein samples were run on a 5-20% SDS-PAGE gel and transferred to a nitrocellulose membrane. The membrane was probed with anti-HA antibody. Protein loading was normalized to the amount of input yeast cells. (**B**) Bimolecular Fluorescence Complementation (BiFC) or split YFP assay. The NH1 protein is fused to the YFP N-terminal half (YN). NRR, W66A (W66), W66A/F70A (WF), and D111A/L112A (DL) are fused to the YFP C-terminal half (YC). When NH1 interacts with NRR, the two halves of YFP are brought together and re-constitute a functional YFP protein, leading to fluorescence. The YFP fluorescence was detected under a fluorescence microscope with a filter set for YFP (excitation: 500 nm; emission: 535 nm).

To confirm the involvement of the NH1-interacting domain in mediating interaction with NH1, we generated two point mutations at conserved amino acids in this domain of NRR and tested their effects on interaction. The first one is a single amino acid mutation changing tryptophan at amino acid 66 to alanine (W66A, labeled as W66); the second one is a double mutation changing phenylalanine at 70 to alanine, in addition to the W66A change (W66A/F70A, labeled as WF). When tested in yeast two-hybrid (Figure 3A), mutation W66A only modestly reduces interaction with NH1. Double mutations WF abolish most of the ability of NRR to interact with NH1. The results of a Western analysis of yeast-expressed B42AD fusion proteins of wild type NRR and mutants W66 and WF (labeled NRR, W66, and WF in the right panel of Figure 3A) probed with an anti-HA antibody show that the W66 and WF fusion proteins accumulate to levels slightly higher than that of wild type NRR (protein loading was normalized to input yeast cell mass). Thus, protein instability can be excluded from being the reason of inability to interact with NH1.

The interaction between NH1 and NRR and these two point mutants were further tested by the Bimolecular Fluorescence Complementation (BiFC) (based on a split YFP assay), which has been successfully used to detect protein-protein interactions in plant cells for many proteins [40-42]. NH1 was fused at its N-terminus to the N-terminal half of the yellow fluorescence protein (YFPN or YN). NRR and mutants W66 and WF were fused at the N-terminus to the C-terminal half of the yellow fluorescence protein (YFPC or YC). In addition, a third mutant was generated which changes amino acids DL at locations 111 and 112 to alanines. The DL amino acids are part of an EAR-motif-like sequence (LDLNxxP), which is a putative transcriptional repression domain shared with NIMIN2, located near the C-terminus of NRR [32]. The pair of proteins was co-expressed transiently in rice protoplasts and fluorescence from the reconstituted YFP protein, when the two proteins interact, was observed semi-quantitatively under a fluorescence microscope.

**Figure 4:**
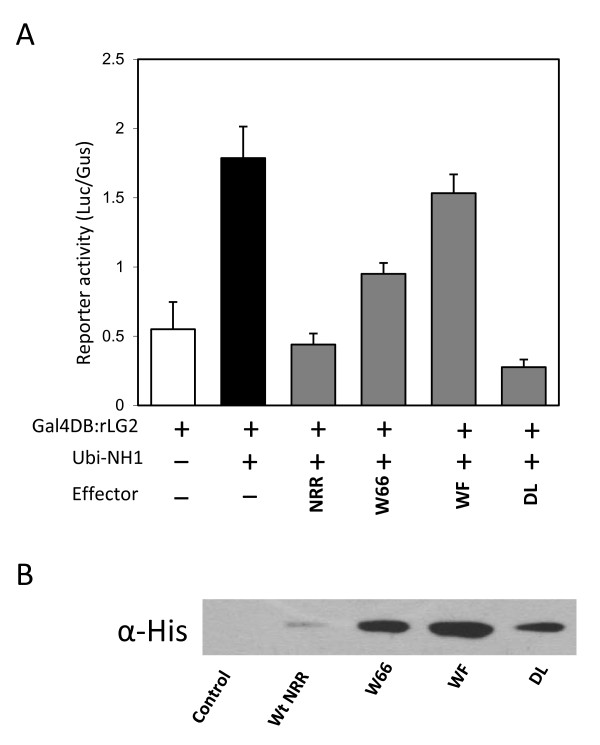
**Effects of the point mutations on the ability of NRR to repress NH1-mediated activation**. (**A**) Protoplast transient assay. The experiment was done as in Figure 1. (**B**) Western blot analysis of total protein extracted from protoplast cells transfected with the different combinations of plasmids. Four replicates of transfection for each combination of plasmids were carried out and the cells combined at the end of transfection. Cells were incubated for 20 h before harvest. The amount of protein loaded for each sample was normalized to the Gus activity expressed from the Ubi-Gus construct included as a reference. Protein on the blot was probed with an anti-His antibody. The wild type NRR protein and mutants W66, WF, and DL are tagged with 6× histidine at the C-terminus.

Representative results of the split YFP experiments are shown in Figure 3B. Figure 3B shows that strong YFP fluorescence was observed from many cells in the wild type NRR-NH1 pair (labeled YC:NRR). Mutant W66 greatly reduces YFP fluorescence intensity and number of positive cells. Double mutant WF further reduces the intensity of fluorescence close to the background level of fluorescence, as observed with the YC negative control. The DL mutant not only fails to reduce the fluorescence, but slightly increases the intensity of YFP fluorescence and the number of positive cells, indicating that it may increase interaction with NH1. This increased interaction could be due to higher affinity or higher protein stability. These in vivo protein-protein interaction results are consistent with the yeast two-hybrid results, confirming the importance of amino acids W66 and F70 in mediating interaction with NH1. The effects of the mutations W66 and WF are not likely due to protein instability because Western analysis of His-tagged NRR and these mutant proteins isolated from rice protoplasts reveal that the mutant proteins are at least as stable as the wild type NRR protein (see Figure 4B).

### Point mutations in the NH1-interacting domain also affect repression on NH1-mediated transcriptional activation

We tested to see if the mutants W66, WF, and DL that affect interaction with NH1 also affect the NH1-mediated transcriptional activation in the rice protoplast transient assay. NH1 activates the rLG-mediated transcription by about 3 fold in this experiment. As shown in Figure 4A, introduction of NRR into the system completely abolishes the NH1-mediated activation. Mutant W66 is less effective than NRR in repressing NH1; double mutant WF has become an ineffective protein in repressing NH1-mediated activation. Interestingly, mutant DL is even more effective in repressing NH1-mediated activation, consistent with the results in Figure 3B, which shows that mutant DL has increased interaction with NH1 than wild type NRR.

We extracted total protein from rice protoplasts transfected with each combination of constructs and probed the NRR protein and its variants with an anti-His antibody against the 6× His-tag on the C-termini of these proteins. The amount of protein loaded in each lane was normalized to the GUS activity, expressed from the *Ubi-Gus *construct included in each transfection as a control for transfection efficiency. Figure 4B shows that a low level of wild type NRR protein accumulates in rice protoplast cells. Mutant W66 and WF proteins accumulate to much higher levels, indicating greater protein stability. Thus, the reduced interaction with NH1 (Figure 3B) and lower effectiveness in repressing NH1-mediated activation (Figure 4A) cannot be due to protein instability. Mutant DL protein also accumulates to a higher level than wild type NRR. This result suggests that the increased interaction of mutant DL with NH1 and more effective repression could be due to higher protein levels resulted from better protein stability. However, an increased affinity for NH1 cannot be ruled out.

### Mutation of the AV sequence in RH2 to ED renders RH2 an effective interactor and repressor of NH1

**Figure 5:**
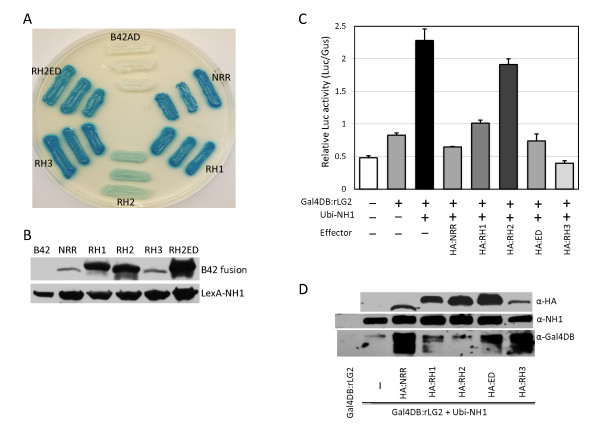
**Effects of the RH2ED mutation on interaction with NH1 and NH1-mediated transcriptional activation**. (**A**) Protein-protein interaction in yeast two-hybrid. Yeast two-hybrid tests were done as described in Figure 3. (**B**) Western analysis of yeast expressed fusion proteins. Protein was extracted from yeast cells containing constructs expressing LexA:NH1 plus B42AD (labeled B42), B42AD:NRR (NRR), B42AD:RH1 (RH1), B42AD:RH2 (RH2), B42AD:RH3 (RH3), or B42AD:RH2ED (RH2ED). Extracted protein samples were run on a 5-20% SDS-PAGE gel and transferred to a nitrocellulose membrane. The membrane was probed with anti-HA and anti-LexA antibodies sequentially. Protein loading was normalized to amount of input yeast cells. (**C**) Effects of the RH2ED mutation on NH1-mediated transcriptional activation. The protoplast transient assay was done as described in Figure 1. (**D**) Western analyses of transiently expressed proteins in protoplasts. Protoplast transfection and protein preparation were done as described in Figure 4. Protein on a nitrocellulose membrane was probed with an anti-HA antibody. A duplicate membrane was probed with anti-NH1 and anti-Gal4DB antibodies sequentially. The amount of protein loaded was normalized to the Gus activity.

To further confirm the amino acid requirement in the NH1-interacting domain, we created a mutant of RH2 (RH2ED) in which the nonconserved AV amino acids are changed to the consensus ED sequence. This RH2ED protein was tested together with NRR, RH1, RH2, and RH3 in yeast two-hybrid for its ability to interact with NH1. As shown in Figure 5A, RH2ED interacts with NH1 as strongly as NRR and RH1 in this assay. RH3 appears to have a higher level of interaction. The levels of these yeast-expressed B42AD fusion proteins were probed with an anti-B42AD antibody and the fusion proteins are labeled as NRR, RH1, RH2, RH3, and RH2ED respectively in Figure 5B. The Western results show that the RH2 protein is stable and accumulates to a level similar to RH1 and higher than NRR and RH3. Thus, the RH2 weak interaction cannot be due to its protein instability. The RH2ED fusion protein accumulates to a level slightly higher than that of RH2. The levels of the LexA:NH1 protein was probed with an anti-LexA antibody and the results show that the LexA:NH1 fusion protein is expressed at similar levels among combinations with NRR, RH1, RH2, RH3 and RH2ED, respectively. These results indicate that the inability of RH2 to interact strongly with NH1 is not due to lower protein levels, but mostly due to the AV sequence deviation.

The RH2ED protein was tested for its ability to repress NH1-mediated activation along with NRR, RH1, RH2, and RH3 in the protoplast transient assay system. In order to readily detect these proteins, an HA tag was introduced at the N-terminus of each protein. Figure 5C shows the results of the transient assay experiments, in which RH2ED (labeled HA:ED) acts as a repressor, as effective as NRR and better than RH1, whereas RH2 remains ineffective in repressing NH1-mediated activation. The levels of these proteins were probed with an anti-HA antibody in Western analyses. Protein loading was normalized to the GUS activity expressed from the *Ubi-Gus *construct included in each transfection as a control. The results (Figure 5D) show that the RH2 protein is stable and accumulates to a level higher than those of NRR, RH1, and RH3. RH2ED accumulates to a level slightly higher than that of RH2. Thus, the difference between RH2 and RH2ED in repressing NH1 is not due to protein levels, but due to the difference in their abilities to interact with NH1.

In addition to probing the effector proteins NRR, RH1, RH2, RH2ED, and RH3, the NH1 protein (untagged) was also probed with an anti-NH1 antibody raised against the N-terminal portion of NH1. The NH1 protein accumulates to a level below the detection threshold without the *Ubi-NH1 *construct and to similar high levels in all reactions containing the *Ubi-NH1 *construct (Figure 5D, α-NH1). The Gal4DB:rLG2 protein was probed with an anti-Gal4DB antibody. Surprisingly, the Gal4DB:rLG2 fusion protein exists at dramatically varying levels (Figure 5D, α-Gal4DB). The Gal4DB:rLG2 fusion protein alone accumulates only to a very low level, barely detectable in our system. Introduction of extra NH1 protein leads to a higher level of the Gal4DB:rLG2 fusion protein. Further introduction of constructs carrying NRR, RH1, RH2ED, and RH3 (in particular NRR, RH2ED, and RH3; labeled HA:NRR, HA:ED, and HA:RH3) greatly increases the Gal4DB:rLG2 protein levels; introduction of RH2 does not significantly increase the Gal4DB:rLG2 protein level. Co-incidentally, NRR, RH2ED, and RH3 are the three most effective proteins in repressing NH1-mediated activation. Therefore, a link between the accumulated rLG2 levels and the repressor effectiveness may exist. Interestingly, degradation of the Gal4DB:rLG2 protein is evident accompanying higher levels of the protein, although the cause remains unclear.

## Discussion

We have used a rice transient assay system based on green rice protoplasts to identify a novel domain in NRR and its family members that is required for strong interaction with rice NH1. Based on available sequence databases, this novel NH1-interacting domain is present in proteins from rice, wheat, maize, sorghum, Populus, and Ricinus, and in two Arabidopsis NIMIN proteins. Thus, this novel NH1-interacting domain is widely present across higher plants, from important cereal crops (rice, wheat, maize. sorghum) to castor bean plants (*Ricinus communis*), to poplar trees (*Populus trichocarpa*), to the model plant Arabidopsis. This family of NH1-interacting domain-containing proteins, represented by rice NRR and NIMINs, mostly likely will interact with NH1-like proteins from the same species that function similarly to NH1 in rice or to NPR1 in Arabidopsis. It is interesting that Arabidopsis NIMIN1 and NIMIN3 also carry a sequence sharing similarity to the NH1-interacting domain even though this sequence is not needed for strong interaction with NPR1. It is puzzling what role this sequence may play in NIMIN1 and NIMIN3. One possibility remains that this conserved sequence in NIMIN1 and NIMIN3 may have undetected, weak interaction with NPR1 and thus may be able to influence the conformation and function of NPR1. This notion remains to be tested. Nevertheless, our protoplast-based transient assay system presents a powerful tool to dissect these proteins.

In our rice protoplast cell transient system, the rTGA2.1 protein functions as a transcriptional repressor, similar to the Arabidopsis TGA2. In contrast, the rLG2 protein functions as a transcriptional activator. This result is surprising and interesting because none of the NPR1-interacting TGA proteins have been shown to act as transcriptional activators in a similar transient assay system in the absence of SA and other proteins, such as NPR1. When NH1 binds to rTGA2.1 or rLG2, it functions as a transcriptional co-activator, similar to Arabidopsis NPR1. Dissimilarly, NPR1 does not activate transcription in transient system until an SAR inducer is added. Thus, it represents a major difference between rice and Arabidopsis systems because NH1 appears to need no ectopic SAR inducers for activation in rice protoplasts. This difference is possibly due to the high endogenous SA content in rice [43]

### How does NRR repress NH1-mediated activation?

Inhibition of NH1-mediated activation is completely correlated with the ability of NRR to bind to NH1. Thus, inhibition of the NH1 activation activity completely depends on the binding ability of NRR. It appears the inhibition is not dependent on the EAR-like motif present near the C-terminus of NRR because mutation of the conserved amino acids DL in this motif failed to abolish inhibition by NRR and because RH1 and RH2 do not contain this EAR-like motif. It is unclear why the NRR protein contains the EAR domain when it is not required for inhibition of NH1. It is possible that NRR may carry out another function, which requires the EAR domain but is not associated with inhibition of NH1.

When NRR binds to NH1, it may keep the NH1 protein in a conformation or state that is unfavorable for interaction with basal transcriptional machinery. For example, NRR may mask the transcriptional activation domain of NH1 or another domain critical for NH1 function. A similar role has been hypothesized for NIMIN1 and NIMIN2 in repressing tobacco NPR1 function based on experiments carried out in yeast [44]. The interaction between NIMINs and tobacco NPR1 is abolished and the repression released upon addition of SA [44]. Alternatively but unlikely, binding of NRR to NH1 may exclude the TGA transcription factors from binding to NH1.

Interestingly, stronger repressors like NRR, RH2ED, and RH3 lead to higher levels of the Gal4DB:rLG protein, while having little effects on the NH1 protein level. These results suggest that the NRR family members may affect the state of the TGA protein through sequestering the TGA protein in a complex and stabilizing it. Alternatively, binding of NRR, RH2ED, and RH3 to NH1 may lead to modifications of the TGA protein, resulting in higher stability. Partial degradation of the Gal4DB:rLG2 protein is evident in these reactions, supporting the notion of modification. In either case, the higher levels of the Gal4DB:rLG protein in the presence of strong NH1-binding repressors, like NRR, RH2ED, and RH3, may be a clue to how these proteins repress TGA-NH1-mediated transcriptional activation.

## Conclusions

The protoplast-based transient system can be used to dissect protein domains associated with their functions. Our results demonstrate that the ability of NRR and its homologues to repress NH1-mediated transcriptional activation is tightly correlated with their ability to bind to NH1. Furthermore, a novel NH1-interacting domain is identified. Importantly, this NH1-interacting domain is widely present in plant species, from cereals to castor bean plants, to poplar trees, and to Arabidopsis, indicating its significance in plants.

## Methods

### Plant materials and protoplast preparation

Rice (*Oryza sativa *L) seeds were surface-sterilized with 30% bleach and germinated and grew on MS (Murashige and Skoog) medium plus 2% sucrose and 0.8% agar in ice cream cone cups in a growth chamber at 28°C with 16 hr lighting. Ten-day old seedlings of UAS-Luc transgenic rice were used for rice protoplast cell preparation according to published protocols [37]. A protoplast cell concentration of 1-5 × 10^6 ^cells/mL was used. For split YFP experiments, Kitaake (Kit) rice plants were grown the same way in dark until ten days old.

### Gene cloning and plasmid construction

For generation of the UAS-promoter construct, oligonucleotides 6xgal-1 (5'AAGAGCTCGG AGTACTGTCC TCCGGAGTAC TGTCCTCCGG AGTACTGTCC TCCGGAGTAC TGTCCTCCGG AGTACTGTCC TCCGGCTATA CGTCTTC3') and 35S-gal (5'AAGGATCCAG CGTGTCCTCT CCAAATGAAA TGAACTTCCT TATATAGAGG AAGGGTCTTG CGAAGGATAG TGGGAAGACG TATAGCCGGA3') were used to assemble the 6x gal4 binding sites-minimal 35S promoter (UAS-35Sp) fragment. This UAS-35Sp fragment was digested with BamHI and SacI and inserted into a Luc/SK plasmid (precut with BamHI/SacI) in front of the Luc gene, generating the UAS-Luc/SK plasmid. The UAS-Luc fragment (including a Nos3') was excised with SacI + HindIII and cloned into binary vector C4300, pre-digested with SacI + HindIII, generating UAS-Luc/C4300. This construct was used to transform Kit rice, yielding UAS-Luc transgenic rice lines.

To make Gal4 fusion constructs Gal4:rTGA2.1 and Gal4:rLG2, the Gal4 DNA binding domain (Gal4DB) was PCR amplified with primers gal4DB-Bam (TTGGATCCAT GAAGCTACTG TCTTCTATC) and gal4DB-E109 (TTCAGGCCCT GCGGCGATAC AGTCAACTGT) and digested with BamHI + Eco01091. The N-terminal of rLG2 (rLG2N) was amplified with primers rLG2-1 (CACCGGTACC GTGATGAGCT CTGTGCGCTA CTG) and rLG2-2 (CATCCACTGA CTTGCCATCT T) and the C-terminal (rLG2C) amplified with primers rLG2-3 (ACTCCAAAGA GCACGGTCAC) and rLG2-4 (TTACTAGTTT CAAAATCCTG AGTACTGATT CTGCTG). rLG2N was digested with KpnI + BamHI and rLG2C was digested with Eco01091 + SpeI. rLG2N, Gal4DB, and rLG2C were jointly cloned into SK-. The Gal4:rLG2 fusion gene was excised with KpnI + SpeI and cloned into modified pENTR/D vector L16 (precut with KpnI + XbaI) and the resulting plasmid was used to recombine the Gal4:rLG2 gene into a Gateway-compatible Ubi-pUC vector, creating construct Ubi-Gal4:rLG2.

The C-terminal of rTGA2.1 (rTGA2.1 C) was amplified with primers mn1-10 (CAGCAGGGCC TCTTCATCTC TAGCTCTGG) and mn1-5 (AAAGGATCCT TACTCCCGTG GCCTAGCAAG), digested with Eco01091 + PstI, and ligated together with Gal4DB to SK- (BamHI + PstI), creating Gal4:rTGA2.1 m/SK. A full-length rTGA2.1 was amplified with primers mn1-ATG (CACCATGGCAGATGCTAGTTCAAGGA) and mn1-5 and cloned into the pENTR/D vector (rTGA2.1f/pENTR). The rTGA2.1f/pENTR was cut with BglII + AscI to release most of the rTGA2.1 gene, and the remaining joined with rTGA2.1 m (BamHI + PstI) and rTGA2.1-3' (PstI + AscI), generating Gal4:rTGA2.1/pENTR. The Gal4:rTGA2.1 gene was recombined into the Ubi-pUC vector, generating Ubi-Gal4:rTGA2.1.

The Ubi-NH1/pUC construct was created through recombination of the NH1 cDNA in the pENTR/D vector into the Gateway-compatible Ubi-pUC vector.

The cDNA clones of NRR, RH1, RH2, and RH3 were amplified with primers NRR-ATG (CACCATGGAC GCCACCACCA CCGCCAAG) + NRR-TAP2 (TTACTAGTTG TAATCCGTGA GCACCCGCAT), RH1-ATG (CACCATGGAG GGAGTTGACG TGAAGGC) + mn133-7 (TTCTCGAGCA AATCAAGACT GGCACATG), RH2-ATG (CACCATGGAA GCCCGATTGA GCACGGG) + 133H-2 (TTTACTAGT CTCGAGCCTG ATTAATTCAT CTGGTCAC), and RH3-ATG (CACCATGGAT CCCACGATGC CCACTCC) + 133H2-3 (TTTACTAGTC TCGAGACTCA TCTGTATGAA CTTG), respectively. Individual cDNA was cloned into the pENTR/D vector and recombined into the Ubi-pUC vector.

### Creation of mutations and addition of epitope tags

Mutation of RH2 to RH2ED was carried out by PCR on the pENTR/D vector containing RH2 cDNA using primers RH2-ED1 (5'GTTCGAGATG GAGGACTTCG AGTGCGG) and RH2-ED2 (5'CACTCGAAGT CCTCCATCTC GAACGC). The mutations were confirmed by sequencing. The HA-tag was introduced into the N-termini of NRR, RH1, RH2, RH2ED, and RH3 via PCR reactions. The primer used to create the HA-tag is ATG-HA (5' CACCATGTAC CCTTACGACG TGCCAGACTA CGCCTCT). Over-lapping 5' primers were used to amplify individual genes containing the HA-tag: NRR-ATGHA (5' GTGCCAGACT ACGCCTCTGA CGCCACCACC ACCGCCAAG), RH1-ATGHA (5' GTGCCAGACT ACGCCTCTGA GGGAGTTGAC GTGAAGGC), RH2-ATGHA (5' GTGCCAGACT ACGCCTCTGA AGCCCGATTG AGCACGGG), and RH3-ATGHA (5' GTGCCAGACT ACGCCTCTGA TCCCACGATG CCCACTCC). Each 5' primer was paired with a 3' primer described above for the individual gene.

Point mutation W66A was generated by PCR using a modified NRR as template with overlapping primers 45-21a (CTCCACGAGC ACCTGCGGCA CGCCCCAGCT TCTCCTG) and 45-22a (CGCAGGTGCT CGTGGAGGGG GAG) and double point mutation with primers 45-21b (CTCCACGAGC ACCTGCGGCA CGCCCCAGCG CATCCTGGGA GGACTTC) and 45-22a. The 6x histidine (His)-tag at the C-terminus of NRR and mutants was introduced by PCR using primer PNI-6Hb (AACTCGAGAC TAGTCAATGG TGATGGTGAT GGTGTGCCGG TGCTCGCGCC GAGCGCGGCG T). Mutant DL was generated by PCR using overlapping primers PNI-DL (GACGGCGGCT CGACGTTAGC TGCGAGGCCG GGAGCAGGT) and PNI-6Hc (TCGCGCCGAG CGCGGCGTGG CCGGCGCGTC GGACGGCGGC TCGACGTT), which overlaps with primer PNI-6Hb. Introduction of the 6xHis tag into mutants W66A, W66A/F70A, and DL was done in the same way.

For transient expression in rice protoplasts, the effector gene was transferred to the expression vector Ubi-pUC by recombination. The individual gene is under control of the maize *Ubi-1 *promoter after recombination.

For yeast two-hybrid assay, 6H-tagged NRR, W66A, and W66A/F70A were cloned into the p42AD vector. The LexA:NH1 construct has been described before. Yeast two-hybrid assay was done as described before [22]. For split YFP assay, NH1 cDNA was recombined into a Gateway-compatible pY736 vector to generate YN:NH1 protein. NRR and its variants were recombined into a Gateway-compatible pY735 vector to generate YC fusion proteins.

### Generation of antibodies against NH1

The 5' end of NH1 cDNA encoding the first 124 amino acids was amplified with primers NH1N-pET1 (5' TTTCATATGGA GCCGCCGACC AGC) and NH1N-pET2 (5' TTGGATCCTA CCCGACCTCC ACCTCCT). The PCR product was digested with NdeI and BamHI and cloned into the pET15b vector via the same cutting sites. The resulting construct NH1N/pET was transferred into E. coli cells BL21. The NH1N peptide was expressed in BL21 cells after induction with 1 mM IPTG (Isopropyl β-D-1-thiogalactopyranoside). The NH1N protein was purified with Ni-NTA resins (Qiagen, Valencia, CA) according the manufacturer's protocol. The NH1N peptide was used to immunize rabbits and raise antibodies against NH1.

### Yellow fluorescence protein (YFP) detection for split YFP assay

Rice protoplasts were incubated for 24-36 h after transfection in incubation buffer. YFP detection used fluorescence microscope Axiovert 25 (Zeiss) with excitation at 500/25 nm and emission at 535/30 nm (filter set 46HE). Pictures were taken with camera Retiga 2000R. Images were not artificially colored.

### Luciferase (Luc) and β-glucuronidase (Gus) activity assays

Luc and Gus activities were assayed as described before [45].

## Competing interests

The authors declare that they have no competing interests.

## Authors' contributions

MC, WB, and PCR designed the study. MC and WB performed most of the experiments and collected the data. LEB assisted in designing primers for the cloning of the UAS-35Sp fragment and in establishing the UAS-Luc transgenic line. PEC performed rice transformation with the UAS-Luc construct. WHS helped carry out genotyping and protoplast transfection. MC and WB drafted the manuscript. MC and PCR carried out the final editing. All authors read and approved the final manuscript.
